# CDDO-Me Attenuates Clasmatodendrosis in CA1 Astrocyte by Inhibiting HSP25-AKT Mediated DRP1-S637 Phosphorylation in Chronic Epilepsy Rats

**DOI:** 10.3390/ijms23094569

**Published:** 2022-04-20

**Authors:** Duk-Shin Lee, Tae-Hyun Kim, Hana Park, Ji-Eun Kim

**Affiliations:** 1 Department of Anatomy and Neurobiology, College of Medicine, Hallym University, Chuncheon 24252, Korea; dslee84@hallym.ac.kr (D.-S.L.); hyun1028@hallym.ac.kr (T.-H.K.); parkhana28@hallym.ac.kr (H.P.); 2 Institute of Epilepsy Research, College of Medicine, Hallym University, Chuncheon 24252, Korea

**Keywords:** 3CAI, AKT, astrocyte, astroglial degeneration, autophagy, epilepsy, HSP25, seizure

## Abstract

Clasmatodendrosis is one of the irreversible astroglial degeneration, which is involved in seizure duration and its progression in the epileptic hippocampus. Although sustained heat shock protein 25 (HSP25) induction leads to this autophagic astroglial death, dysregulation of mitochondrial dynamics (aberrant mitochondrial elongation) is also involved in the pathogenesis in clasmatodendrosis. However, the underlying molecular mechanisms of accumulation of elongated mitochondria in clasmatodendritic astrocytes are elusive. In the present study, we found that clasmatodendritic astrocytes showed up-regulations of HSP25 expression, AKT serine (S) 473 and dynamin-related protein 1 (DRP1) S637 phosphorylations in the hippocampus of chronic epilepsy rats. 2-Cyano-3,12-dioxo-oleana-1,9(11)-dien-28-oic acid methyl ester (CDDO-Me; bardoxolone methyl or RTA 402) abrogated abnormal mitochondrial elongation by reducing HSP25 upregulation, AKT S473- and DRP1 S637 phosphorylations. Furthermore, HSP25 siRNA and 3-chloroacetyl-indole (3CAI, an AKT inhibitor) abolished AKT-DRP1-mediated mitochondrial elongation and attenuated clasmatodendrosis in CA1 astrocytes. These findings indicate that HSP25-AKT-mediated DRP1 S637 hyper-phosphorylation may lead to aberrant mitochondrial elongation, which may result in autophagic astroglial degeneration. Therefore, our findings suggest that the dysregulation of HSP25-AKT-DRP1-mediated mitochondrial dynamics may play an important role in clasmatodendrosis, which would have implications for the development of novel therapies against various neurological diseases related to astroglial degeneration.

## 1. Introduction

Temporal lobe epilepsy (TLE) is one of the common neurological diseases, which is characterized by presence of spontaneous episodes of abnormal excessive neuronal discharges [1,2]. Neuronal loss including γ-aminobutyric acid (GABA)-ergic interneurons and synaptic rearrangement lead to seizure generation (ictogenesis) and the development of epilepsy (epileptogenesis). Together with the dysfunctions of neurons, aberrant astroglial functionality also contributes to pathogenesis of TLE, since astrocytes control the homeostasis of synaptic transmission and blood brain barrier (BBB), and glia-induced inflammation [2,3,4,5]. Indeed, astroglial dysfunctions such as disturbance of astrocyte gap junction coupling and K^+^ buffering are involved in the etiology of TLE [6]. Although astrocytes are believed to be resistant to harmful stresses [7,8], astroglial degeneration is also induced by various pathological conditions [5,9,10,11]. In particular, clasmatodendrosis (an irreversible autophagic astroglial death) has been reported by Alzheimer and Cajal more than 100 years ago [12]. Clasmatodendritic astrocytes show extensive swollen cell bodies with lysosome-derived vacuoles indicating ubiquitin proteasome system (UPS)-mediated autophagocytosis and disintegrated/beaded processes [12,13,14,15,16]. Furthermore, this astroglial degeneration may be involved in the synchronous epileptiform discharges and regulate seizure duration, but not seizure on-set or its severity, in chronic epilepsy rats [11,17].

The underlying molecular mechanisms of clasmatodendrosis are closely relevant to dysregulations of extracellular signal-related kinases 1/2 (ERK1/2)-specificity protein 1 (SP1)-heat shock protein 25 (HSP25)-mediated endoplasmic reticulum (ER) stress, which hyperactivates AKT and facilitates autophagic process independent of mammalian target of rapamycin (mTOR) activity [18,19,20,21,22,23]. Furthermore, mitochondrial defects initiated by acidosis and aberrant mitochondrial dynamics are involved in clasmatodendritic astroglial degeneration [24,25,26,27,28]. 

Mitochondria are highly dynamic organelles, which play important role in the production of adenosine triphosphate (ATP), cellular homeostasis, Ca^2+^ regulation, and reactive oxygen species (ROS) generation [29,30]. Therefore, mitochondria change morphologies to maintain their functions properly in response to cellular energetic status via two opposing processes: fusion and fission (referred as mitochondrial dynamics). Mitochondrial fusion increases mitochondrial length to maintain and restore mitochondrial function by facilitating the stochastic redistribution of soluble and membrane components between normal and defective mitochondria. Fission shortens mitochondrial length for the elimination of irreversibly damaged mitochondria via mitophagy. Imbalance of mitochondrial dynamics induces mitochondrial dysfunctions and subsequently cell death. For example, aberrant enlarged (giant) mitochondria show swelling, loss of cristae and destruction of the inner membrane, indicating mitochondrial functional deficiencies such as decreased ATP production [31,32,33,34]. Mitochondrial dynamics are regulated by various molecules including mitofusin 1 (MFN1), MFN2, optic atrophy 1 (OPA1), dynamin-related protein 1 (DRP1), fission related protein-1 (Fis-1) and mitochondrial fission factor (MFF) [31,32,33]. Among them, DRP1 is a key player for mitochondrial fission, whose activity is differently regulated by phosphorylation at distinct sites. Phosphorylation of DRP1 at serine (S) 616 by cyclin dependent kinase (CDK) 1/Cyclin B, CDK5 or ERK1/2 promotes mitochondrial fission, while DRP1-S637 phosphorylation by protein kinase A (PKA) detaches DRP1 from mitochondria and inhibits mitochondrial fission. Therefore, maintenance of DRP1 S616/S637 phosphorylation ratio is tightly regulated for cell viability [35,36,37,38]. Indeed, the reduced DRP1-S616/S637 phosphorylation ratio leads to aberrant mitochondrial elongations in clasmatodendritic astrocytes within the stratum radiatum of CA1 region (CA1 astrocytes) without altering other mitochondrial dynamics-related molecules [28]. Interestingly, AKT directly phosphorylates DRP1 S637 site [39,40,41]. Therefore, it is likely that upregulated AKT phosphorylation may affect DRP1-mediated mitochondrial dynamics during clasmatodendrosis, which has been elusive. 

2-Cyano-3,12-dioxo-oleana-1,9(11)-dien-28-oic acid methyl ester (CDDO-Me; bardoxolone methyl or RTA 402) is a derivatives of synthetic triterpenoids and has anti-inflammatory, antioxidant, anti-proliferative properties [42]. Since CDDO-Me inhibits HSP25-induced AKT S473 phosphorylation in clasmatodendritic CA1 astrocytes [17], it is likely that CDDO-Me may also attenuate clasmatodendrosis by recovering the impairment of DRP1-mediated mitochondrial dynamics in CA1 astrocytes. In the present study, therefore, we furthermore explored the effect of CDDO-Me on clasmatodendrosis to elucidate how clasmatodendritic events would evoke abnormal mitochondrial elongations, in spite of activations of autophagic process, and which signaling pathway would be involved in the dysregulations of mitochondrial dynamics during clasmatodendrosis.

Here, we demonstrate that CDDO-Me ameliorated clasmatodendrosis in CA1 astrocytes in the hippocampus of chronic epilepsy rats. In addition, CDDO-Me decreased accumulation of elongated mitochondria in CA1 astrocytes, concomitant with the HSP25 downregulation and the reduced DRP1 S637 and AKT S473 phosphorylation levels, which increased the DRP1-S616/S637 phosphorylation ratio. HSP25 knockdown showed the similar effect on clasmatodendritic CA1 astrocytes. 3-Chloroacetyl-indole (3CAI, an AKT inhibitor [43]) also mitigated clasmatodendrosis without altering prolonged HSP25 upregulation in CA1 astrocytes. Therefore, our findings suggest that sustained HSP25 induction may trigger the impaired mitochondrial fission in CA1 astrocytes during clasmatodendrosis by enhancing AKT-mediated DRP1 S637 phosphorylation, which was mitigated by CDDO-Me.

## 2. Results

### 2.1. CDDO-Me Attenuates HSP25-Mediated Autophagy in CA1 Astrocytes

**Figure 1:**
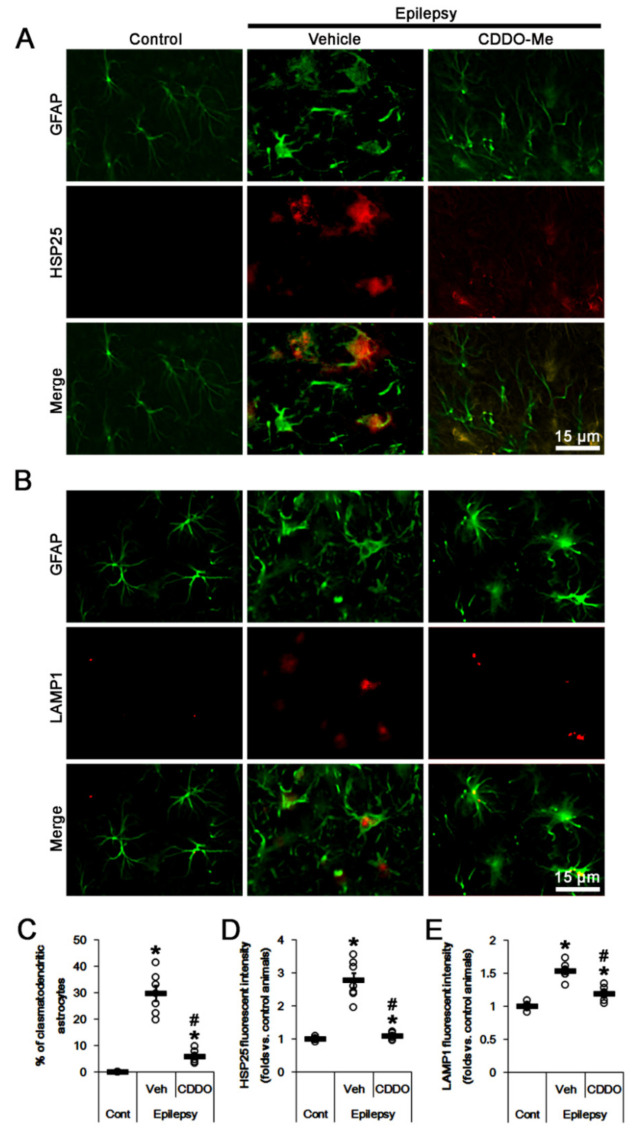
Effects of CDDO-Me on heat shock protein 25 (HSP25) and lysosomal-associated membrane protein 1 (LAMP1) expressions in CA1 astrocytes of control and epileptic rats. As compared to control animals (Cont), HSP25 and LAMP1 expressions are upregulated in CA1 astrocytes in epileptic rats. CDDO-Me reduces the increased HSP25 and LAMP1 expressions in CA1 astrocytes, as compared to vehicle (Veh). (**A**,**B**) Representative photos demonstrating astroglial HSP25 (**A**) and LAMP1 (**B**) expressions in CA1 astrocytes. (**C**–**E**) Quantifications of the fraction of clasmatodendritic astrocytes in total CA1 astrocytes (**C**), HSP25 expression (**D**) and LAMP1 fluorescent intensity (**E**) in CA1 astrocytes. Open circles indicate each value. Horizontal bars indicate the mean value. Error bars indicate SEM (*, ^#^ *p* < 0.05 vs. control and vehicle-treated epileptic rats, respectively; *n*  = 7, respectively).

In chronic epilepsy rats (8 weeks after SE), 30% of CA1 astrocytes showed typical clasmatodendrosis, which showed strong HSP25 and lysosomal-associated membrane protein 1 (LAMP1) positive vacuoles (*t*_(12)_ = 10.2, *p* < 0.001 vs. control animals, Student *t*-test, *n* = 7, respectively; Figure 1A–C). The fluorescent intensities of HSP25 and LAMP1 were 2.78 (*t*_(12)_ = 8, *p* < 0.001 vs. control animals, Student *t*-test, *n* = 7, respectively; Figure 1A,D) and 1.53-fold of control level (*t*_(12)_ = 10.1, *p* < 0.001 vs. control animals, Student *t*-test, *n* = 7, respectively; Figure 1B,E). In CDDO-Me-treated animals, most of CA1 astrocytes were typical reactive forms showing swelling and hypertrophy without vacuolization (Figure 1A,B). CDDO-Me decreased the fraction of clasmatodendritic astrocytes in CA1 astrocytes to 6% (*F*_(2,18)_ = 81.1, *p* < 0.001, one-way ANOVA, *n* = 7, respectively; Figure 1A–C), and reduced the fluorescent intensities of HSP25 and LAMP1 to 0.55 (*F*_(2,18)_ = 44.8, *p* < 0.001, one-way ANOVA, *n* = 7, respectively; Figure 1A,D) and 0.78-fold of vehicle-treated animal level (*F*_(2,18)_ = 60, *p* < 0.001, one-way ANOVA, *n* = 7, respectively; Figure 1B,E). Consistent with our previous study [17], these findings indicate that CDDO-Me may attenuate clasmatodendritic CA1 astroglial degeneration in the epileptic hippocampus.

### 2.2. CDDO-Me Reduces AKT S473 Phosphoprylation and Mitochondrial Length in CA1 Astrocytes

**Figure 2:**
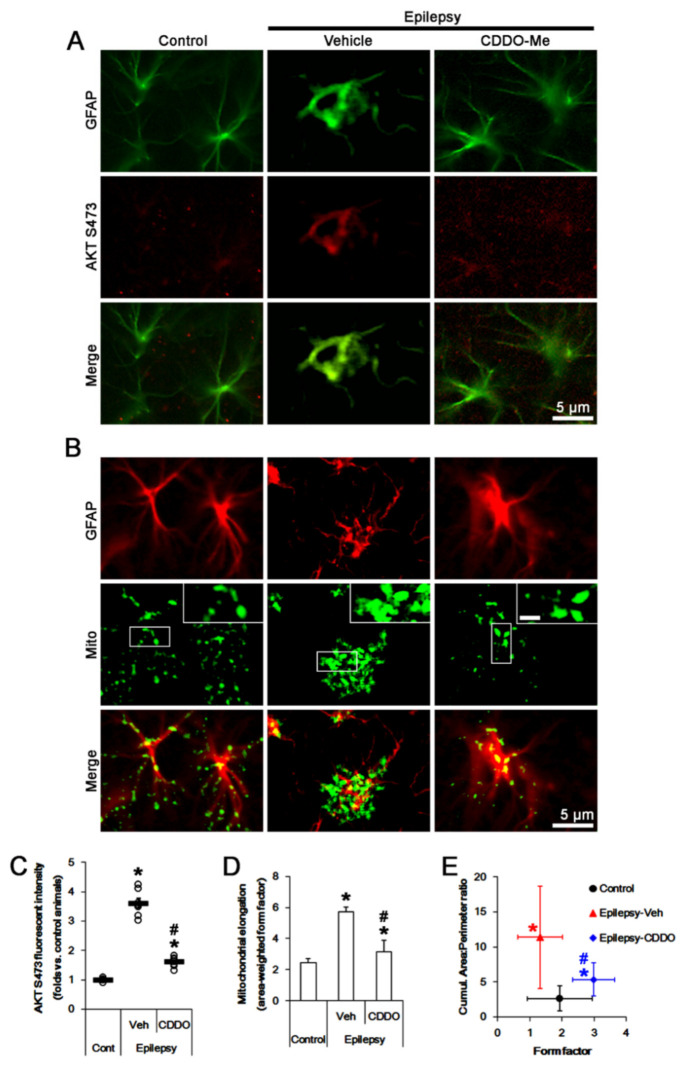
Effects of CDDO-Me on AKT S473 phosphorylation and mitochondrial length in CA1 astrocytes of control and epileptic rats. As compared to control animals (Cont), AKT S473 phosphorylation and mitochondrial length are increased in CA1 astrocytes in epileptic rats. CDDO-Me reduces AKT S473 phosphorylation and mitochondrial length in CA1 astrocytes, as compared to vehicle (Veh). (**A**,**B**) Representative photos demonstrating astroglial AKT S473 phosphorylation (**A**) and mitochondrial morphology (**B**) in CA1 astrocytes. (**C**–**E**) Quantifications of AKT S473 phosphorylation (mean ± S.E.M.; *, ^#^ *p* < 0.05 vs. control and vehicle-treated epileptic rats, respectively; *n* = 7, respectively, (**C**)), mitochondrial elongation index (area-weighted form factor, mean ± S.E.M.; *, *^#^ p* < 0.05 vs. control and vehicle-treated epileptic rats, respectively; *n* = 7, respectively, (**D**)) and the cumulative area:perimeter ratio (an indicative of the transition from elongated, isolated mitochondria to a reticular network or aggregation of interconnected mitochondria) and the form factor (a parameter as transition from the sphere to elongated, complex shaped, but still isolated mitochondria; mean ± S.E.M.; *, ^#^ *p* < 0.05 vs. control and vehicle-treated epileptic rats, respectively; *n* = 7, respectively, (**E**)).

The dysregulation of mitochondrial dynamics is one of the causes for clasmatodendrosis in CA1 astrocytes. Briefly, aberrant mitochondrial elongation evokes clasmatodendritic (autophagic) degeneration in CA1 astrocytes [28]. Considering the inhibitory effect of CDDO-Me on clasmatodendrosis in CA1 astrocytes [17] and the AKT-mediated regulation of mitochondrial dynamics [39,40,41], it is likely that CDDO-Me may rescue aberrant mitochondrial elongation (fusion) by inhibiting AKT activity. To confirm this, we evaluate the effect of CDDO-Me on AKT activity (phosphorylation). In chronic epilepsy rats, clasmatodendritic CA1 astrocytes showed the upregulated AKT S473 fluorescent intensity and the accumulation of elongated mitochondria (Figure 2A,B). The AKT S473 fluorescent intensity was 3.61-fold of control level (*t*_(12)_ = 15.5, *p* < 0.001 vs. control animals, Student *t*-test, *n* = 7, respectively; Figure 2A,C). 

In control animals, astroglial mitochondrial elongation (area-weighted form factor [44,45,46]) was 2.43 (Figure 2A,D). The cumulative area:perimeter ratio (an indicative of the transition from elongated, isolated mitochondria to a reticular network or aggregation of interconnected mitochondria [44,45]) and the form factor (a parameter as transition from the sphere to elongated, complex shaped, but still isolated mitochondria [44,45,46]) were 1.94 and 2.64, respectively (Figure 2B,E). In chronic epilepsy rats, astroglial mitochondrial elongation was 5.7 (*t*_(68)_ = 5.6, *p* < 0.001 vs. control animals, Student *t*-test, *n* = 7, respectively; Figure 2A,D). The cumulative area:perimeter ratio and the form factor were 11.37 (*t*_(68)_ = 8.9, *p* < 0.001 vs. control animals, Student *t*-test, *n* = 7, respectively) and 1.33 (*t*_(68)_ = 3.8, *p* < 0.001 vs. control animals, Student *t*-test, *n* = 7, respectively), respectively (Figure 2B,E), indicating the accumulation of elongated mitochondria. 

CDDO-Me decreased AKT S473 fluorescent intensity to 0.44-fold of vehicle-treated animal level (*F*_(2,18)_ = 170, *p* < 0.001, one-way ANOVA, *n* = 7, respectively; Figure 2A,C). CDDO-Me reduced mitochondrial elongation to 3.13 (*F*_(2,102)_ = 6.1, *p* = 0.003, one-way ANOVA, *n* = 7, respectively; Figure 2B,D) and the cumulative area:perimeter ratio to 5.34 (*F*_(2,102)_ = 12.2, *p* < 0.001, one-way ANOVA, *n* = 7, respectively; Figure 2B,E). However, CDDO-Me increased the form factor to 2.99 (*F*_(2,102)_ = 21.7, *p* < 0.001, one-way ANOVA, *n* = 7, respectively; Figure 2B,E). These findings indicate that CDDO-Me may abrogate the accumulation of elongated mitochondria in CA1 astrocytes, accompanied by the reduced AKT activity. 

### 2.3. CDDO-Me Reduces DRP1 S637 Phosphorylation in CA1 Astrocytes without Affecting S616 Phosphorylation

DRP1 oppositely regulates mitochondrial dynamics by two distinct sites: Phosphorylation at S616 is associated with increased activity of DRP1 (profission), whereas phosphorylation at S637 is linked to reduced activity (antifission) [35,36,37,38]. Thus, we investigated the effects of CDDO-Me on DRP1 S616 and S637 phosphorylations.

**Figure 3:**
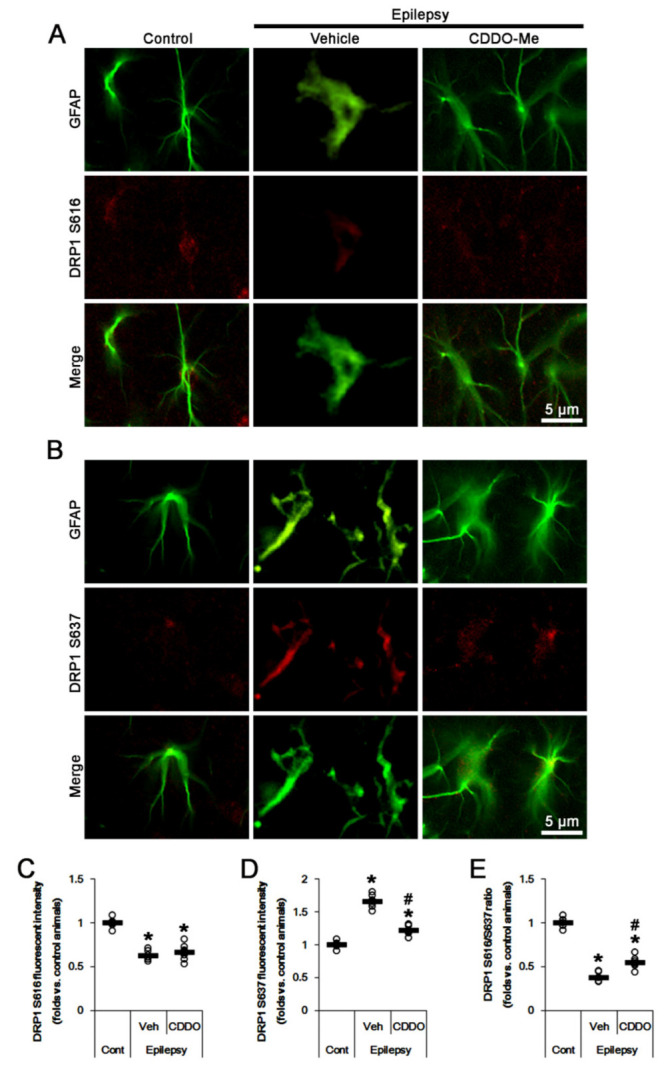
Effects of CDDO-Me on DRP1 S616- and S637 phosphorylations in CA1 astrocytes of control and epileptic rats. As compared to control animals (Cont), DRP1 S616 phosphorylation is reduced in CA1 astrocytes in epileptic rats, while DRP1 S637 phosphorylation is increased. CDDO-Me reduces the increased DRP1 S637 phosphorylation in CA1 astrocytes without affecting DRP1 S616 phosphorylation, as compared to vehicle (Veh). (**A**,**B**) Representative photos demonstrating astroglial DRP1 S616- (**A**) and S637 phosphorylation (**B**) in CA1 astrocytes. (**C**–**E**) Quantifications of DRP1 S616- (**C**), S637 phosphorylation (**D**) and DRP1 S616/S637 phosphorylation ratio (**E**) in CA1 astrocytes. Open circles indicate each value. Horizontal bars indicate the mean value. Error bars indicate SEM (*, # *p* < 0.05 vs. control and vehicle-treated epileptic rats, respectively; *n* = 7, respectively).

In chronic epilepsy rats, DRP1 S616 phosphorylation was lower than that in control animals, while the S637 phosphorylation was higher (Figure 3A–D). The DRP1 S616 fluorescent intensity was 0.62-fold of control level (*t*_(12)_ = 13.1, *p* < 0.001 vs. control animals, Student *t*-test, *n* = 7, respectively; Figure 3A,C), while DRP1 S637 fluorescent intensity was 1.66-fold of control level (*t*_(12)_ = 15.5, *p* < 0.001 vs. control animals, Student *t*-test, *n* = 7, respectively; Figure 3B,D). Thus, DRP1 S616/S637 ratio was reduced to 0.38-fold of control level (*t*_(12)_ = 21.7, *p* < 0.001 vs. control animals, Student *t*-test, *n* = 7, respectively; Figure 3E). Although CDDO-Me did not affect DRP1 S616 fluorescent intensity, it decreased S637 fluorescent intensity to 0.73-fold of vehicle-treated animal level (*F*_(2,18)_ = 138.3, *p* < 0.001, one-way ANOVA, *n* = 7, respectively; Figure 3A–D). Therefore, DRP1 S616/S637 ratio was elevated to 1.45-fold of vehicle-treated animal level (*F*_(2,18)_ = 206.8, *p* < 0.001, one-way ANOVA, *n* = 7, respectively; Figure 3E). These findings indicate that CDDO-Me may attenuate aberrant mitochondrial elongation by inhibiting DRP1 S637 phosphorylation in clasmatodendritic CA1 astrocytes. 

### 2.4. HSP25 Knockdown Inhibits AKT S473 and DRP1 S637 Phosphorylations in CA1 Astrocytes

**Figure 4:**
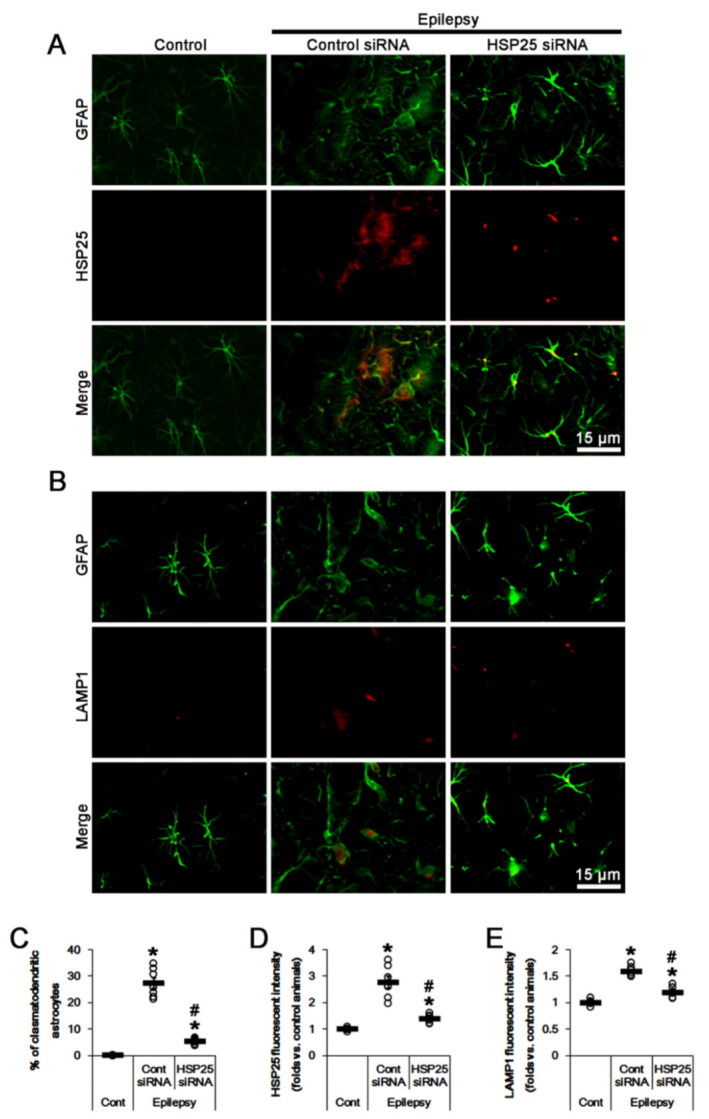
Effects of HSP25 knockdown on heat shock protein 25 (HSP25) and lysosomal-associated membrane protein 1 (LAMP1) expressions in CA1 astrocytes of control and epileptic rats. As compared to control animals (Cont), HSP25 and LAMP1 expressions are upregulated in CA1 astrocytes in epileptic rats. HSP25 siRNA reduces the increased HSP25 and LAMP1 expressions in CA1 astrocytes, as compared to control siRNA (cont siRNA). (**A**,**B**) Representative photos demonstrating astroglial HSP25 (**A**) and LAMP1 (**B**) expressions in CA1 astrocytes. (**C**–**E**) Quantifications of the fraction of clasmatodendritic astrocytes in total CA1 astrocytes (**C**), HSP25 expression (**D**) and LAMP1 fluorescent intensity (**E**) in CA1 astrocytes. Open circles indicate each value. Horizontal bars indicate the mean value. Error bars indicate SEM (*, ^#^ *p* < 0.05 vs. control and control siRNA-treated epileptic rats, respectively; *n* = 7, respectively).

To confirm whether sustained HSP25 induction directly affects AKT S473 and DRP1 S637 phosphorylations in clasmatodendritic CA1 astrocytes, we applied HSP25 knockdown in chronic epilepsy rats. Most of CA1 astrocytes showed typical reactive forms in HSP25 siRNA-infused animals (Figure 4A,B). HSP25 siRNA decreased the fraction of clasmatodendritic astrocytes in CA1 astrocytes to 5.3% (*F*_(2,18)_ = 147.4, *p* < 0.001, one-way ANOVA, *n* = 7, respectively; Figure 4A–C), and reduced the fluorescent intensities of HSP25 and LAMP1 to 0.51 (*F*_(2,18)_ = 47.2, *p* < 0.001, one-way ANOVA, *n* = 7, respectively; Figure 4A,D) and 0.75-fold of control siRNA-treated animal level in chronic epilepsy rats (*F*_(2,18)_ = 83.4, *p* < 0.001, one-way ANOVA, *n* = 7, respectively; Figure 4B,E). 

**Figure 5:**
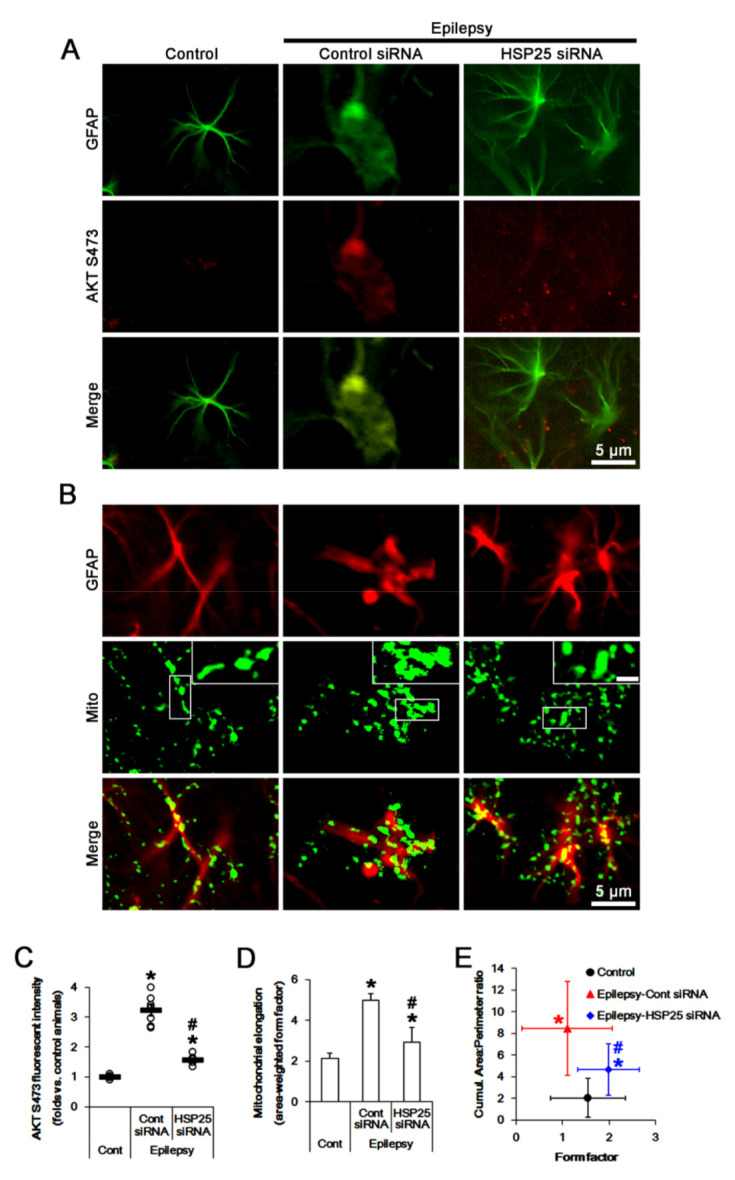
Effects of HSP25 knockdown on AKT S473 phosphorylation and mitochondrial length in CA1 astrocytes of control and epileptic rats. As compared to control animals (Cont), AKT S473 phosphorylation and mitochondrial length are increased in CA1 astrocytes in epileptic rats. HSP25 siRNA reduces AKT S473 phosphorylation and mitochondrial length in CA1 astrocytes, as compared to control siRNA (cont siRNA). (**A**,**B**) Representative photos demonstrating astroglial AKT S473 phosphorylation (**A**) and mitochondrial morphology (**B**) in CA1 astrocytes. (**C**–**E**) Quantifications of AKT S473 phosphorylation (mean ± S.E.M.; *, # *p* < 0.05 vs. control and control siRNA-treated epileptic rats, respectively; *n* = 7, respectively, (**C**)), mitochondrial elongation index (area-weighted form factor, mean ± S.E.M.; *, # *p* < 0.05 vs. control and control siRNA-treated epileptic rats, respectively; *n* = 7, respectively, (**D**)) and the cumulative area:perimeter ratio (an indicative of the transition from elongated, isolated mitochondria to a reticular network or aggregation of interconnected mitochondria) and the form factor (a parameter as transition from the sphere to elongated, complex shaped, but still isolated mitochondria; mean ± S.E.M.; *, # *p* < 0.05 vs. control and control siRNA-treated epileptic rats, respectively; *n* = 7, respectively, (**E**)).

HSP25 knockdown decreased AKT S473 fluorescent intensity to 0.49-fold of control siRNA-treated animal level (*F*_(2,18)_ = 105.2, *p* < 0.001, one-way ANOVA, *n* = 7, respectively; Figure 5A,C). In control siRNA-treated animals, astroglial mitochondrial elongation was 4.99 (Figure 5B,D). The cumulative area:perimeter ratio and the form factor were 8.47 and 1.1 (Figure 5B,E). HSP25 siRNA reduced mitochondrial elongation to 2.93 (*F*_(2,102)_ = 9.4, *p* < 0.001, one-way ANOVA, *n* = 7, respectively; Figure 5B,D) and the cumulative area:perimeter ratio to 4.64 (*F*_(2,102)_ = 14.8, *p* < 0.001, one-way ANOVA, *n* = 7, respectively; Figure 5B,E). HSP25 siRNA increased the form factor to 1.99 (*F*_(2,102)_ = 9.7, *p* < 0.001, one-way ANOVA, *n* = 7, respectively; Figure 5B,E). 

**Figure 6:**
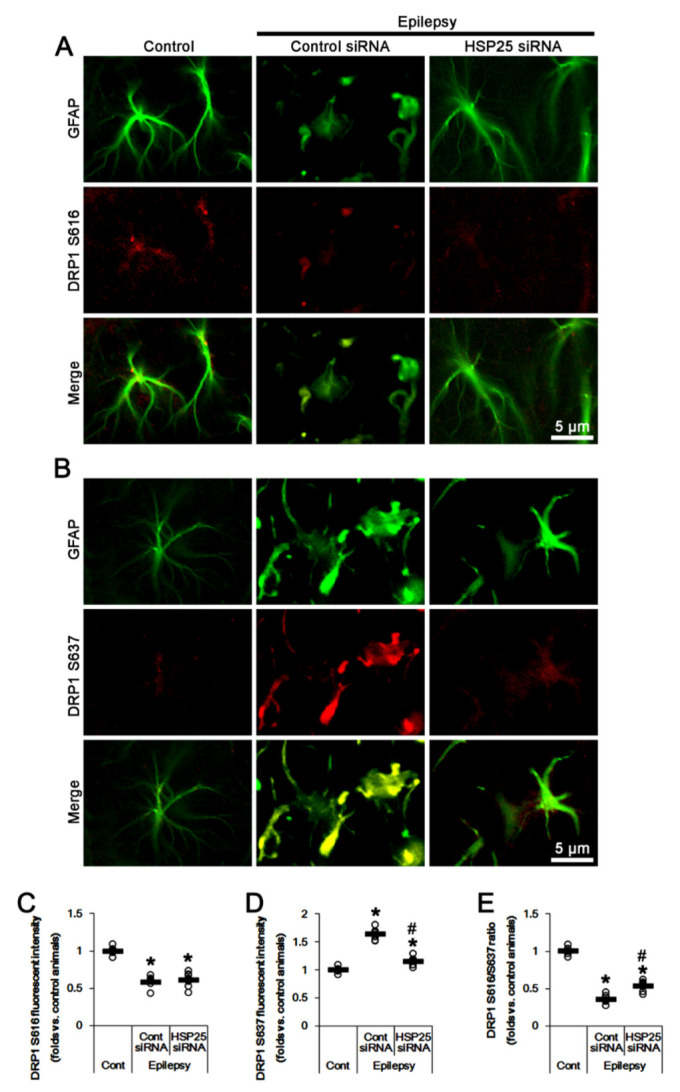
Effects of HSP25 knockdown on DRP1 S616- and S637 phosphorylations in CA1 astrocytes of control and epileptic rats. As compared to control animals (Cont), DRP1 S616 phosphorylation is reduced in CA1 astrocytes in epileptic rats, while DRP1 S637 phosphorylation is increased. CDDO-Me reduces the increased DRP1 S637 phosphorylation in CA1 astrocytes without affecting DRP1 S616 phosphorylation, as compared to control siRNA (cont siRNA). (**A**,**B**) Representative photos demonstrating astroglial DRP1 S616- (**A**) and S637 phosphorylation (**B**) in CA1 astrocytes. (**C**–**E**) Quantifications of DRP1 S616- (**C**), S637 phosphorylation (**D**) and DRP1 S616/S637 phosphorylation ratio (**E**) in CA1 astrocytes. Open circles indicate each value. Horizontal bars indicate the mean value. Error bars indicate SEM (*, ^#^ *p* < 0.05 vs. control and control siRNA-treated epileptic rats, respectively; *n* = 7, respectively).

HSP25 siRNA knockdown did not affect DRP1 S616 fluorescent intensity in CA1 astrocytes (Figure 6A,C). However, HSP25 knockdown decreased S637 fluorescent intensity to 0.7-fold of control siRNA-treated animal level (*F*_(2,18)_ = 114.2, *p* < 0.001, one-way ANOVA, *n* = 7, respectively; Figure 6B,D). Thus, DRP1 S616/S637 ratio was elevated to 1.47-fold of control siRNA-treated animal level (*F*_(2,18)_ = 206.2, *p* < 0.001, one-way ANOVA, *n* = 7, respectively; Figure 6E). Taken together, these findings indicate that prolonged HSP25 induction may evoke the accumulation of elongated mitochondria in CA1 astrocytes by increasing AKT S473 and DRP1 S637 phosphorylations.

### 2.5. 3CAI Decreases AKT S473 and DRP1 S637 Phosphorylations in CA1 Astrocytes

**Figure 7:**
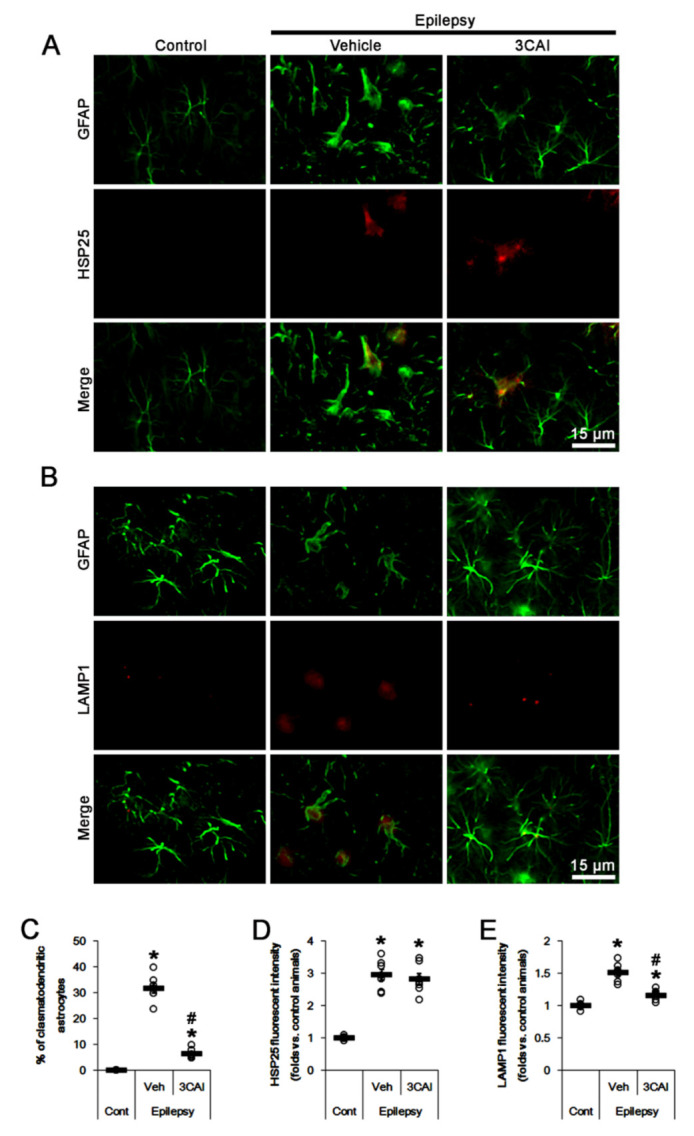
Effects of 3CAI on heat shock protein 25 (HSP25) and lysosomal-associated membrane protein 1 (LAMP1) expressions in CA1 astrocytes of control and epileptic rats. As compared to control animals (Cont), HSP25 and LAMP1 expressions are upregulated in CA1 astrocytes in epileptic rats. 3CAI reduces the increased LAMP1, but not HSP25, expression in CA1 astrocytes, as compared to vehicle (Veh). (**A**,**B**) Representative photos demonstrating astroglial HSP25 (**A**) and LAMP1 (**B**) expressions in CA1 astrocytes. (**C**–**E**) Quantifications of the fraction of clasmatodendritic astrocytes in total CA1 astrocytes (**C**), HSP25 expression (**D**) and LAMP1 fluorescent intensity (**E**) in CA1 astrocytes. Open circles indicate each value. Horizontal bars indicate the mean value. Error bars indicate SEM (*, ^#^ *p* < 0.05 vs. control and vehicle-treated epileptic rats, respectively; *n* = 7, respectively).

To investigate directly the role of AKT in DRP1 S637 phosphorylation in CA1 astrocytes, we applied 3CAI (an AKT inhibitor) in chronic epilepsy rats. 3CAI reduced the fraction of clasmatodendritic astrocytes in CA1 astrocytes to 6.4% (*F*_(2,18)_ = 208.8, *p* < 0.001, one-way ANOVA, *n* = 7, respectively; Figure 7A–C). Thus, most of CA1 astrocytes showed typical reactive in 3CAI-infused animals (Figure 7A,B). However, 3CAI did not affect HSP25 fluorescent intensity, but decreased LAMP1 fluorescent intensity to 0.77-fold of vehicle-treated animal level in CA1 astrocytes (*F*_(2,18)_ = 49.9, *p* < 0.001, one-way ANOVA, *n* = 7, respectively; Figure 7A–E).

**Figure 8:**
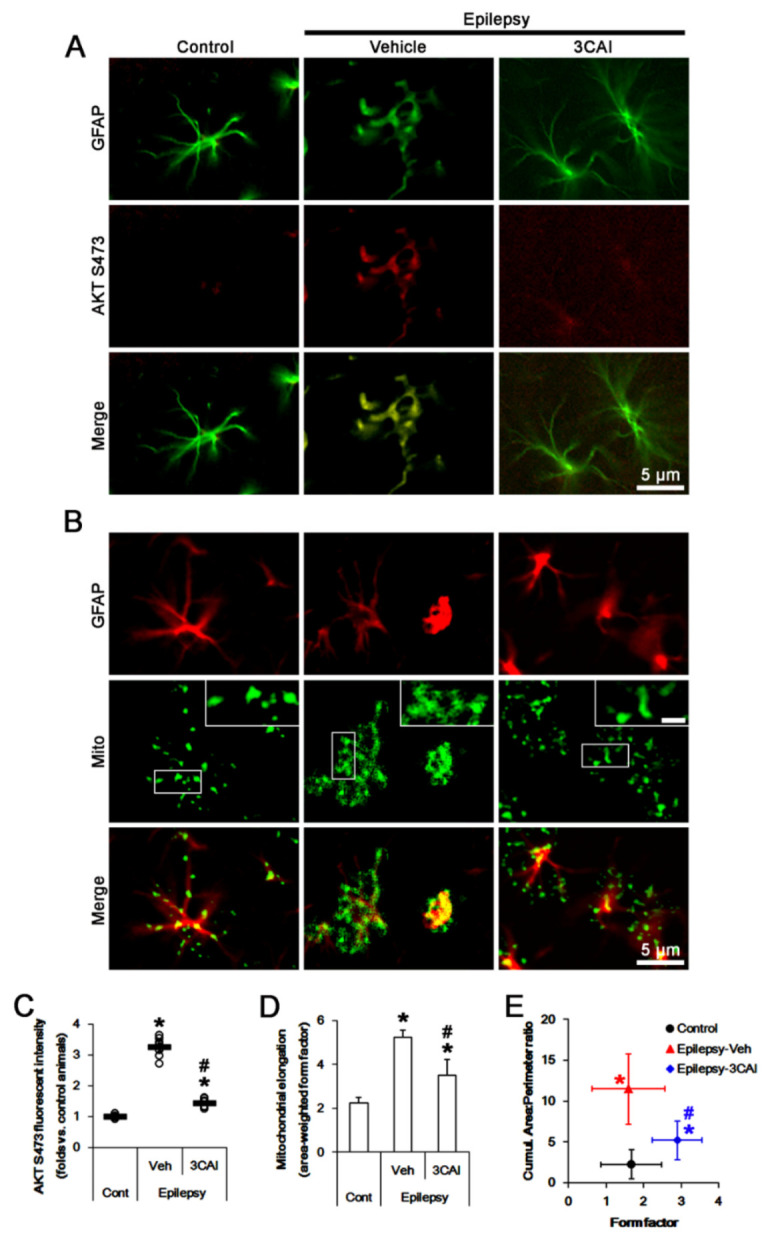
Effects of 3CAI on AKT S473 phosphorylation and mitochondrial length in CA1 astrocytes of control and epileptic rats. As compared to control animals (Cont), AKT S473 phosphorylation and mitochondrial length are increased in CA1 astrocytes in epileptic rats. 3CAI reduces AKT S473 phosphorylation and mitochondrial length in CA1 astrocytes, as compared to vehicle (Veh). (**A**,**B**) Representative photos demonstrating astroglial AKT S473 phosphorylation (**A**) and mitochondrial morphology (**B**) in CA1 astrocytes. (**C**–**E**) Quantifications of AKT S473 phosphorylation (mean ± S.E.M.; *, ^#^ *p* < 0.05 vs. control and vehicle-treated epileptic rats, respectively; *n*  = 7, respectively, (**C**)), mitochondrial elongation index (area-weighted form factor, mean ± S.E.M.; *, ^#^ *p* < 0.05 vs. control and vehicle-treated epileptic rats, respectively; *n* = 7, respectively, (**D**)) and the cumulative area:perimeter ratio (an indicative of the transition from elongated, isolated mitochondria to a reticular network or aggregation of interconnected mitochondria) and the form factor (a parameter as transition from the sphere to elongated, complex shaped, but still isolated mitochondria; mean ± S.E.M.; *, ^#^ *p* < 0.05 vs. control and vehicle-treated epileptic rats, respectively; *n* = 7, respectively, (**E**)).

3CAI diminished AKT S473 fluorescent intensity to 0.44-fold of vehicle-treated animal level (*F*_(2,18)_ = 224.7, *p* < 0.001, one-way ANOVA, *n* = 7, respectively; Figure 8A,C). In vehicle-treated animals, astroglial mitochondrial elongation was 5.23 (Figure 8B,D). The cumulative area:perimeter ratio and the form factor were 11.47 and 1.6 (Figure 8B,E). 3CAI reduced mitochondrial elongation to 3.5 (*F*_(2,102)_ = 8.3, *p* < 0.001, one-way ANOVA, *n* = 7, respectively; Figure 8B,D) and the cumulative area:perimeter ratio to 5.2 (*F*_(2,102)_ = 15.3, *p* < 0.001, one-way ANOVA, *n* = 7, respectively; Figure 8B,E). 3CAI increased the form factor to 2.89 (*F*_(2,102)_ = 10.4, *p* = 0.001, one-way ANOVA, *n* = 7, respectively; Figure 8B,E).

**Figure 9:**
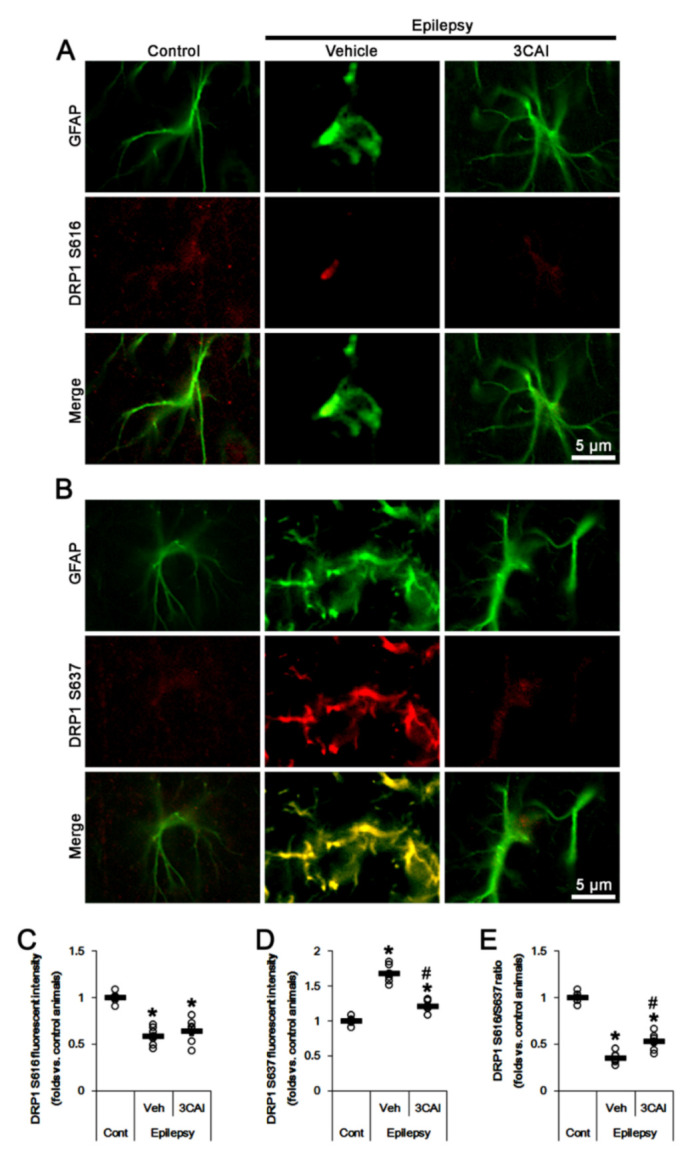
Effects of 3CAI on DRP1 S616- and S637 phosphorylations **in** CA1 astrocytes of control and epileptic rats. As compared to control animals (Cont), DRP1 S616 phosphorylation is reduced in CA1 astrocytes in epileptic rats, while DRP1 S637 phosphorylation is increased. 3CAI reduces the increased DRP1 S637 phosphorylation in CA1 astrocytes without affecting DRP1 S616 phosphorylation, as compared to vehicle (Veh). (**A**,**B**) Representative photos demonstrating astroglial DRP1 S616- (**A**) and S637 phosphorylation (**B**) in CA1 astrocytes. (**C**–**E**) Quantifications of DRP1 S616- (**C**), S637 phosphorylation (**D**) and DRP1 S616/S637 phosphorylation ratio (**E**) in CA1 astrocytes. Open circles indicate each value. Horizontal bars indicate the mean value. Error bars indicate SEM (*, ^#^ *p* < 0.05 vs. control and vehicle-treated epileptic rats, respectively; *n* = 7, respectively).

3CAI reduced S637 fluorescent intensity to 0.72-fold of vehicle-treated animal level in CA1 astrocytes (*F*_(2,18)_ = 108.3, *p* < 0.001, one-way ANOVA, *n* = 7, respectively) without affecting DRP1 S616 fluorescent intensity (Figure 9A–D). In addition, 3CAI increased DRP1 S616/S637 ratio to 1.37-fold of vehicle-treated animal level (*F*_(2,18)_ = 164.9, *p* < 0.001, one-way ANOVA, *n* = 7, respectively; Figure 9E). Therefore, our findings indicate that upregulated AKT activity may result in the aberrant mitochondrial elongation in clasmatodendritic CA1 astrocytes by DRP1 S637 hyper-phosphorylation.

### 2.6. CDDO-Me and 3CAI Decrease DRP1 S637, but Not S616, Phosphorylations in CA1 Astrocytes

**Figure 10:**
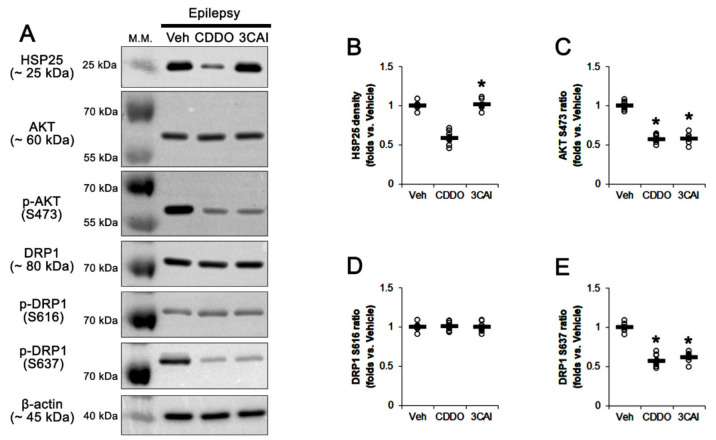
Effects of CDDO-Me and 3CAI on HSP25, AKT S473, DRP1 S616 and DRP1 S637 levels in the stratum radiatum of the CA1 region of epileptic rats. As compared to vehicle (Veh), CDDO-Me (CDDO) reduces HSP25, AKT S473 and DRP1 S637 levels. 3CAI decreases AKT S473 and DRP1 S637 levels. (**A**) Representative Western blot images of HSP25, AKT, AKT S473, DRP1, DRP1 S616 and DRP1 S637. (**B**–**E**) Quantifications of HSP25 (**B**), AKT S473 (**C**), DRP1 S616 (**D**) and DRP1 S637 levels. Open circles indicate each value. Horizontal bars indicate the mean value. Error bars indicate SEM (* *p* < 0.05 vs. Vehicle, respectively; *n* = 7, respectively).

To confirm the effects of CDDO-Me on DRP1 phosphorylation, we performed the Western blot using the stratum radiatum of the CA1 region where astrocytes were mainly observed. Consistent with our previous study [17], CDDO-Me decreased HSP25 (*t*_(12)_ = 9.99, *p* < 0.001, Student *t*-test, *n* = 7, respectively), AKT S473 (*t*_(12)_ = 13.68, *p* < 0.001, Student *t*-test, *n* = 7, respectively) and DRP1 S637 level (*t*_(12)_ = 10.75, *p* < 0.001, Student *t*-test, *n* = 7, respectively; Figure 10 and Supplementary Figure S1). 3CAI reduced AKT S473 (*t*_(12)_ = 12.53, *p* < 0.001, Student *t*-test, *n* = 7, respectively) and DRP1 S637 phosphorylation (*t*_(12)_ = 11.98, *p* < 0.001, Student *t*-test, *n* = 7, respectively; Figure 10 and Supplementary Figure S1) without altering HSP25 level. Both CDDO-Me and 3CAI did not affect DRP1 S616 phosphorylation (Figure 10 and Supplementary Figure S1). Together with immunohistochemical data, our findings indicate that CDDO-Me may attenuate autophagic astroglial degeneration by inhibiting HSP25-AKT-DRP1 signaling pathway.

## 3. Discussion

Clasmatodendrosis is the type II programmed astroglial death induced by excessive or unquenched autophagic process [21,22,47,48]. Clasmatodendrosis is observed in patients of traumatic brain injury, Alzheimer’s disease, cerebrovascular accidents and mixed dementia [15,49,50]. This irreversible astroglial degeneration is regulated by various signaling pathways including ERK1/2, AKT, 5′ adenosine monophosphate-activated protein kinase (AMPK) and P2X7 receptor [17,21,23]. On the other hand, the accumulation of elongated mitochondria also leads to autophagic degeneration of CA1 astrocytes, although OPA1 (a mitochondrial fusion protein) expression is reduced in clasmatodendritic astrocytes of chronic epilepsy rats. Indeed, mitochondrial division inhibitor 1 (Mdivi-1) accelerates and exacerbates clasmatodendritic changes in CA1 astrocytes [28]. However, the underlying mechanisms of dysregulation of mitochondrial dynamics in clasmatodendritic astrocytes are poorly understood.

Although HSP25 plays a protective role against harmful stress, prolonged HSP25 translation results in high-energy consumption and ER stress and finally triggers astroglial autophagy [18,20,22]. Compatible with a previous study [17], the present data demonstrate that CDDO-Me may ameliorate clasmatodendrosis by inhibiting dysregulation of HSP25-AKT signaling pathway. HSP25 modulates AKT enzyme activity, since HSP25 acts as a chaperone to retain AKT S473 phosphorylation by abrogating the pleckstrin homology domain and leucine-rich repeat protein phosphatase (PHLPP) 1- and 2-binding to AKT. Therefore, sustained HSP25 induction is sufficient for AKT-mediated astroglial autophagy, although HSP25 is not an indispensable factor for AKT activation [21,23,51,52]. Indeed, the present study shows that both CDDO-Me and HSP25 siRNA attenuated clasmatodendrosis, accompanied by the reduced AKT S473 phosphorylation. Therefore, our findings suggest that HSP25-AKT signaling pathway may play a key role in clasmatodendritic astroglial degeneration.

AKT directly binds with DRP1 and phosphorylates S637 site. This AKT-mediated DRP1 S637 phosphorylation leads to the impaired mitochondrial fission (mitochondrial elongation) by DRP1 inactivation [39,53]. In the present study, clasmatodendritic CA1 astrocytes showed the accumulation of elongated mitochondria, concomitant with the increased AKT S473 and DRP1 S637 phosphorylations. Furthermore, CDDO-Me, HSP25 siRNA and 3CAI rescued clasmatodendrosis and aberrant mitochondrial elongation by reducing DRP1 S637 phosphorylation. Therefore, these findings indicate that HSP25-AKT-mediated DRP1 S637 hyper-phosphorylation may impair mitochondrial fission, which subsequently may provoke abnormal mitochondrial elongation in clasmatodendritic astrocytes.

CDDO-Me prevents prolonged HSP25 induction by enhancing ERK1/2 activity that would also phosphorylate DRP1 S616 site [54,55]. Indeed, DRP1 activation through S616 phosphorylation is regulated by ERK/AKT [56]. In addition, amyloid-β (Aβ) sustains AKT activation that induces DRP1 S616 phosphorylation, and facilitates mitochondrial fission in neurons [57]. Ca^2+^ influx induced by Aβ activates Ca^2+^/calmodulin-dependent protein kinase II (CaMKII)-AKT signaling pathway, which facilitates DRP1-mediated mitochondrial fragmentations and suppresses mammalian target of rapamycin (mTOR)-dependent autophagy in neurons [57]. However, clasmatodendrosis is mTOR-independent astroglial autophagy that is regulated by HSP25-mediated AKT activation [21,23]. Furthermore, the present data reveal that CDDO-Me did not affect DRP1 S616 phosphorylation, and that clasmatodendritic astrocytes showed the accumulation of elongated mitochondria and the AKT S473 hyper-phosphorylation. These findings indicate that CDDO-Me-induced ERK1/2 activation may not be involved in DRP1-mediated mitochondrial fission, but may inhibit the prolonged HSP25 induction in clasmatodendritic astrocytes. In addition, the increased AKT activity in clasmatodendritic astrocytes may elongate mitochondrial length by enhancing DRP1 phosphorylation at S637 rather than S616 site. Therefore, it is plausible that the distinct signaling pathways in response to the disparate stimuli would cause the different DRP1 regulations.

The dysregulation of mitochondrial fission (aberrant mitochondrial elongation) leads to oxidative stress and further elevates reactive oxygen species, which triggers autophagic cell death [58,59,60,61]. In the present study, CDDO-Me abrogated the aberrant mitochondrial elongation in clasmatodendritic CA1 astrocytes by increasing DRP1 S616/S637 phosphorylation ratio. CDDO-Me is an activator of nuclear factor-erythroid 2-related factor 2 (Nrf2, a redox-sensitive transcription factor) that maintains redox homeostasis by regulating antioxidant-response element (ARE)-dependent transcription and the expression of antioxidant defense enzymes including heme oxygenase-1 (HO-1), which protect neurons and astrocytes from various harmful stresses [17,38,55,62,63]. Therefore, it is presumable that the antioxidant effect of CDDO-Me would restore the abnormal mitochondrial elongation, and subsequently inhibit clasmatodendrosis in CA1 astrocytes. However, the present study shows that CDDO-Me inhibited AKT that promotes HO-1 gene expression in rat astrocytes [64]. Furthermore, AKT inactivation by 3CAI abrogated accumulation of elongated mitochondria by reducing DRP1 S637 phosphorylation. Given the deterioration of autophagic astroglial death induced by Mdivi-1 [28], therefore, our findings indicate that CDDO-Me may attenuate clasmatodendrosis by direct regulation of AKT-mediated DRP1 phosphorylation as well as its antioxidant properties.

Astrocytes play an important role in delays clearance of K^+^ and glutamate from extracellular space [11,65,66]. Therefore, astroglial dysfunction or death is involved in spontaneous seizure activity and epileptogenesis [5,11,17,67,68]. Clasmatodendrosis is a consequence of spontaneous recurrent seizures due to over-activation of the temporoammonic path, which is involved in the duration and propagation of synchronous discharges (but not its frequency and severity) in the epileptic hippocampus [11,17]. Furthermore, conventional anti-epileptic drugs prevent clasmatodendrosis in chronic epilepsy rats [11]. Although CDDO-Me and 3CAI have not anti-epileptic properties [54,69], furthermore, CDDO-Me attenuates reduces seizure duration and its progression, accompanied by abrogating clasmatodendritic degeneration [17], and 3CAI improves the efficacies of α-Amino-3-hydroxy-5-methylisoxazole-4-propionic acid receptor (AMPAR) antagonists on spontaneous seizure activities [43]. Considering that α-aminoadipic acid (an astroglial toxin) and 4-aminopyridine (a K^+^ channel blocker) synchronizes reverberant epileptiform discharges [11,17,67], clasmatodendrosis may be one of considering factors leading to prolonged seizure activity and its propagation in the epileptic hippocampus through impairments of inwardly K^+^ channel as well as GJ, although it may not be a primary cause of ictogenesis. Therefore, the prevention of clasmatodendrosis may attenuate the duration and propagation of synchronous discharges in the epileptic hippocampus by exerting clearance of K^+^ and glutamate from extracellular space in ictal stage.

CDDO-Me has also anti-cancer properties. Indeed, CDDO-Me induces autophagy, apoptosis and endoplasmic reticulum (ER) stress with the increased ERK1/2 activity and the suppression of AKT/mTOR signaling pathway in human chronic myeloid leukemia K562 cells [70]. Unlike cancer cells, CDDO-Me increases astroglial viability (attenuates clasmatodendrosis) by relieving dysregulation of ERK1/2-mediated HSP25 and aberrant AKT hyperactivation [17,21]. These discrepancies would be relevant to the dose of CDDO-Me applied in the studies. Nanomolar doses of CDDO-Me protect cells against oxidative stress by activating Nrf2. In vivo cell protective dose of CDDO-Me are ~0.05 nmol/kg/day intracerebroventricular (i.c.v.) infusion over 7 days [54,71] and 0.4–4 μmol/kg once-intravenous injection (i.v.) [62,63]. In contrast, the anti-cancer (cytotoxic) concentrations of CDDO-Me in vivo are extremely higher than cell protective concentration. In vivo anti-cancer doses of CDDO-Me are at least 15 μmol/kg/day per oral (p.o.) over 7 or 20 weeks [72,73,74]. Thus, it is likely that the dose of CDDO-Me applied in the present study (~0.05 nmol/kg/day, i.c.v.) could avoid the adverse (cytotoxic) effects of CDDO-Me.

On the other hand, inflammation-related disturbance and dysregulation of astroglial gap junction connexin 43 (Cx43) contribute to the seizure generation, because the uncoupling of Cx43 results in gliotransmitter release and the accumulation of K^+^ and glutamate in the extracellular space [75,76]. Interestingly, carbenoxolone (a gap junction blocker) attenuated astroglial swelling, rupture of astroglial nuclear membrane and vacuolization of the astroglial cytoplasm at post-SE 60 days [76]. Therefore, it is possible that CDDO-Me would ameliorate clasmatodendrosis by affecting Cx43 functionality. Further studies are needed to elucidate the CDDO-Me-induced Cx43 regulation.

## 4. Materials and Methods

### 4.1. Experimental Animals and Chemicals

Adult male Sprague-Dawley (SD) rats (7 weeks old) were used in the present study. Animals were kept under controlled environmental conditions (23–25 °C, 12 h light/dark cycle) to access freely to water and food throughout the experiments. All experimental protocols described below were approved by the Institutional Animal Care and Use Committee of Hallym University (Chuncheon, South Korea, Code number: Hallym 2018-3, approval date: 30 April 2018). All reagents were obtained from Sigma-Aldrich (St. Louis, MO, USA), except as noted.

### 4.2. Epilepsy Model

Animals were subjected to the LiCl-pilocarpine model of temporal lobe epilepsy (TLE). Rats were given LiCl (127 mg/kg, i.p.) 24 h before the pilocarpine treatment. Animals were treated with pilocarpine (30 mg/kg, i.p.) 20 min after atropine methylbromide (5 mg/kg i.p.). Two hours after status epilepticus (SE) onset, diazepam (Valium; Hoffmann-la Roche, Neuilly-sur-Seine, France; 10 mg/kg, i.p.) was administered to terminate SE and repeated, as needed. Control animals received saline in place of pilocarpine. Animals were video-monitored 8 h a day for selecting chronic epileptic rats showing spontaneous recurrent seizures [17,22,43].

### 4.3. Surgery for CDDO-Me, HSP25 or 3CAI Infusion

Epileptic rats (7 weeks after SE) were implanted with a brain infusion kit 1 pump (Alzet, Cupertino, CA, USA) into the right lateral ventricle (1 mm posterior; 1.5 mm lateral; −3.5 mm depth to the bregma) under Isoflurane anesthesia (3% induction, 1.5–2% for surgery, and 1.5% maintenance in a 65:35 mixture of N_2_O:O_2_), and connected with an Alzet 1007D osmotic pump (Alzet, Cupertino, CA, USA) to infuse with (1) vehicle, (2) CDDO-Me (10 μM), (3) 3CAI (25 μM), (4) non-targeting control siRNA (5-GCAACUAACUUCGUUAGAAUCGUUAUU-3) or (5) HSP25 siRNA (5-CUUGGCUCCAGACUGUUCCUU-3). The pump was placed in a subcutaneous pocket in the dorsal region. Each compound (siRNA) was infused over 7-day period. Electrode and infusion needle were secured to the exposed skull with dental acrylic [17,22,43].

### 4.4. Tissue Processing and Immunohistochemistry

Seven days after infusion (8 weeks after SE), animals were transcardially perfused with 4% paraformaldehyde under urethane anesthesia (1.5 g/kg i.p.), and after additional fixation for overnight at 4 °C. The brains were rinsed in PB containing 30% sucrose at 4 °C for 2 days. Thereafter, coronal sections (30 μm) were cut with a cryostat. Age-matched control (normal) animals were also perfused by the same method. Then sections were incubated in 0.1% bovine serum albumin and subsequently primary antibody (Table 1). Tissue sections visualized with appropriate Cy2- and Cy3-conjugated secondary antibodies. Immunofluorescence was observed using an Axio Scope microscope (Carl Zeiss Korea, Seoul, South Korea). Negative control test was performed with normal rabbit serum (#31883, ThermoFisher Korea, Seoul, South Korea), mouse IgG1 isotype control (#02-6100, ThermoFisher Korea, Seoul, South Korea), and mouse IgG2a isotype control (#02-6200, ThermoFisher Korea, Seoul, South Korea), instead of the primary antibodies. No immunoreactivity was observed for the negative control in any structures [17,22,43].

### 4.5. Western Blots

Animals were sacrificed via decapitation. The brains were quickly removed and coronally cut to 1 mm thickness using rodent brain matrix (World Precision Instruments, Sarasota, FL, USA) on ice. Thereafter, the stratum radiatum of the CA1 region of the dorsal hippocampus were dissected out in cold artificial cerebrospinal fluid (4 °C) under stereomicroscope. The tissues were were homogenized and protein concentration determined using a Micro BCA Protein Assay Kit (Pierce Chemical, Rockford, IL, USA). Following electrophoresis, proteins were transferred to nitrocellulose membranes that were blocked overnight with 2% bovine serum albumin in Tris-buffered saline (in mM 10 Tris, 150 NaCl, pH 7.5, and 0.05% Tween 20) and then incubated overnight at 4 °C in blocking solution containing primary antibodies (Table 1). After washing, membranes were incubated for 1 h at room temperature in a solution containing horseradish peroxidase-conjugated secondary antibodies. A chemiluminescence signal was detected by luminol substrate reaction (ECL Western Blotting System, GE Healthcare Korea, Seoul, South Korea). The bands were detected and quantified on an ImageQuant LAS4000 system (GE Healthcare Korea, Seoul, South Korea). The values of each sample were normalized with the corresponding amount of β-actin. The ratio of phosphoprotein to total protein was described as phosphorylation level [17,22,43].

### 4.6. Cell Count, Measurement of Fluorescent Intensity and Mitochondrial Morphometry

The hippocampal tissues were captured (10 sections per each animal), and areas of interest (1 × 10^5^ μm^2^) were selected from the striatum radiatum of the CA1 region. Thereafter, clasmatodendritic astrocytes were counted on 20× images using AxioVision Rel. 4.8 Software. In addition, five brain sections from each animal were randomly selected at different rostro-caudal hippocampal levels. One randomly selected CA1 astrocytes (naïve astrocytes in control animals, and clasmatodendritic and reactive astrocytes in epileptic animals) from each slice (total 35 cells in each group, respectively) were used for quantification of mitochondrial morphometry using ImageJ software. Mitochondria were analyzed for perimeter and area. Mitochondrial parameters were calculated as followed: Area-weighted form factor = perimeter^2^/4π (an indicative of mitochondrial elongation); Form factor = perimeter^2^/4π × area (indicating the transition from punctiform to elongated, complex shaped, but still isolated mitochondria); Cumulative area:perimeter ratio = Σarea/Σperimeter (indicating the transition from elongated, isolated mitochondria to a reticular network or aggregation of interconnected mitochondria) [44,45,46]. For measurement of fluorescent intensity, 30 areas/rat (400 μm^2^/area) were randomly selected within the stratum radiatum of CA1 region (15 sections from each animal, *n* = 7 in each group). Mean intensity was measured using AxioVision Rel. 4.8 software (Carl Zeiss Korea, Seoul, South Korea). Fluorescent intensity was normalized by setting the mean background [44,45,46]. The investigators were blinded to experimental groups in performing cell counts and morphological analysis.

### 4.7. Data Analysis

Comparisons of data among groups were performed using Student *t*-test or one-way ANOVA followed by Bonferroni’s *post hoc* comparisons after evaluating the values on normality using Shapiro-Wilk *W*-test. A *p*-value less than 0.05 was considered to be significant.

## 5. Conclusions

The present data demonstrate for the first time that HSP25-mediated AKT activation impaired mitochondrial fission by DRP1 S637 hyper-phosphorylation and led to autophagic astroglial degeneration, which was abrogated by CDDO-Me, HSP25 siRNA and 3CAI. These new findings may have implications for the development of novel therapies against various neurological diseases by regulating astroglial degeneration and mitochondrial dynamics.

## Figures and Tables

**Table 1:** Primary antibodies used in the present study.

Antigen	Host	Manufacturer (Catalog Number)	Dilution Used
AKT	Rabbit	Cell signaling (#9272)	1:1000 (WB)
AKT S473	Rabbit	Cell Signaling (#4060)	1:100 (IF) 1:1000 (WB)
DRP1	Rabbit	Thermo (PA1-16987)	1:1000 (WB)
DRP1 S616	Rabbit	Cell Signaling (#4494)	1:500 (IF) 1:1000 (WB)
DRP1 S637	Rabbit	Cell Signaling (#4867)	1:500 (IF) 1:1000 (WB)
GFAP	Rabbit Mouse	Abcam (#ab7260) Millipore (#MAB3402)	1:500 (IF) 1:2000 (IF)
HSP25	Rabbit	Enzo (#ADI-SPA-801)	1:500 (IF) 1:1000 (WB)
LAMP1	Rabbit	Lifespan (#LS-B580)	1:200 (IF)
Mitochondrial marker (MTCO1)	Mouse	Abcam (#ab14705)	1:500 (IF)

IF, Immunofluorescence; WB, Western blot.

## Data Availability

Not applicable.

## Supplementary Materials

The following supporting information can be downloaded at: https://www.mdpi.com/article/10.3390/ijms23094569/s1.

## Abbreviations

3CAI	3-chloroacetyl-indole
AMPAR	α-amino-3-hydroxy-5-methylisoxazole-4-propionic acid receptor
ARE	antioxidant-response element
ATP	adenosine triphosphate
Aβ	amyloid-β
BBB	blood brain barrier
CaMKII	Ca^2+^/calmodulin-dependent protein kinase II
CDDO-Me	2-cyano-3,12-dioxo-oleana-1,9(11)-dien-28-oic acid methyl ester
CDK	cyclin dependent kinase
DRP1	dynamin-related protein 1
ER	endoplasmic reticulum
ERK1/2	extracellular signal-related kinases 1/2
Fis-1	fission related protein-1
GABA	γ-aminobutyric acid
GFAP	glial fibrillary acidic protein
HO-1	heme oxygenase-1
HSP25	heat shock protein 25
LAMP1	lysosomal-associated membrane protein 1
MFF	mitochondrial fission factor
MFN1	mitofusin 1
mTOR	mammalian target of rapamycin
Nrf2	nuclear factor-erythroid 2-related factor 2
OPA1	optic atrophy 1
PHLPP	pleckstrin homology domain and leucine-rich repeat protein phosphatase
PKA	protein kinase A
ROS	reactive oxygen species
SD	Sprague-Dawley
SP1	specificity protein 1
TLE	temporal lobe epilepsy
UPS	ubiquitin proteasome system.
