# Supplementary material for: CDDO-Me Attenuates Clasmatodendrosis in CA1 Astrocyte by Inhibiting HSP25-AKT Mediated DRP1-S637 Phosphorylation in Chronic Epilepsy Rats
Source: Int J Mol Sci. 2022 Apr 20;23(9):4569. doi: 10.3390/ijms23094569 (PMC9105539; doi:10.3390/ijms23094569)
Supplement: Supplementary file 1 [file ijms-23-04569-s001.zip › ijms-1671130-supplementary.pdf]

Supplementary Information

CDDO-Me attenuates clasmatodendrosis in CA1 astrocyte by inhibiting HSP25-AKT mediated DRP1-S637 phosphorylation in chronic epilepsy rats

Duk-Shin Lee[1,2], Tae-Hyun Kim[1,2], Hana Park[1,2] and Ji-Eun Kim[1,2]*

[1] Department of Anatomy and Neurobiology, College of Medicine, Hallym University, Chuncheon 24252, Korea
[2] Institute of Epilepsy Research, College of Medicine, Hallym University, Chuncheon 24252, Korea
* Correspondence: jieunkim@hallym.ac.kr; Tel.:+82-33-248-2522; Fax: +82-33-248-2525

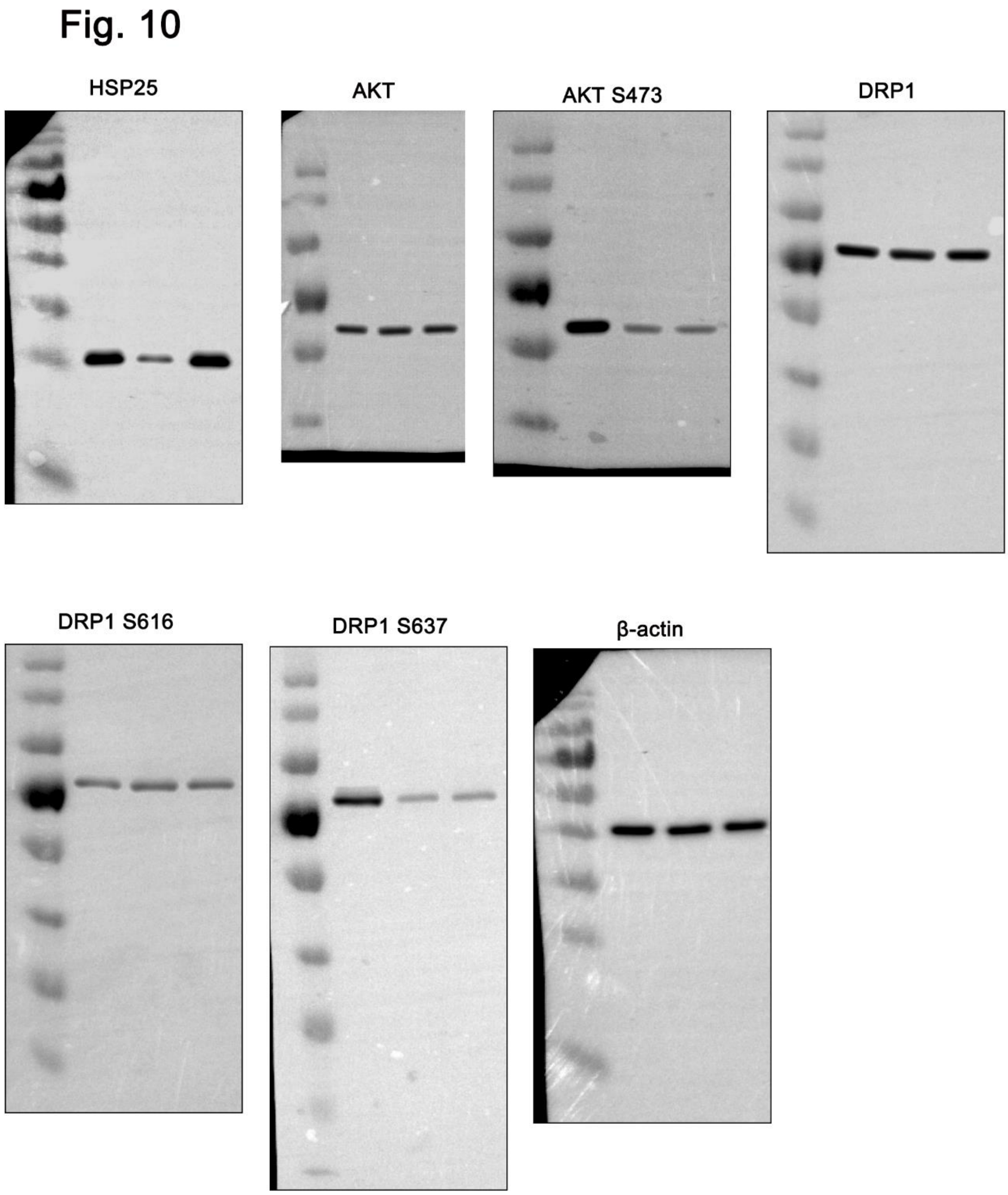

Supplementary Figure S1. Full-length gel images of Western blot data in Figure 10A.
